# Validation and clinical relevance of footprint anatomical masking in clubfoot

**DOI:** 10.1186/1757-1146-7-S1-A25

**Published:** 2014-04-08

**Authors:** Claudia Giacomozzi, Julie Stebbins, Louise Way

**Affiliations:** 1 Department of Technology and Health, Istituto Superiore di Sanità, Rome, Italy; 2 Nuffield Oxford Orthopaedic Centre, Oxford, UK

## Background

Anatomy-based regionalization of pressure dynamic footprints has been proved to be feasible when accurate kinematic and baropodometric measurements are integrated [1]. The potential of this method is easily understandable when footprints are incomplete or severely altered; however, its thorough validation on healthy and pathologic feet is still required. This study focusses on anatomy-based masking in paediatric clubfoot using the Oxford Foot Model (OFM, [2]), which identifies 5 plantar regions of high clinical relevance in this population. Validation is based on the comparison with traditional geometrical masking using the same 5 regions, applied to young healthy volunteers and clubfeet.

## Materials and methods

19 healthy volunteers (H: mean age 11.5 years, mean BMI 18.1) and 10 patients with clubfoot (P: mean age 10.8 years, mean BMI 19.9) were examined at the Oxford Gait Lab by using the OFM and an integrated experimental setup based on a VICON motion system and an EMED-m baropodometer. 3-5 footprints per foot were acquired for each individual while walking barefoot at self-selected speed. Markers projection onto the dynamic footprint allowed the anatomical identification (AM) of: medial hindfoot (M01), lateral hindfoot (M02), midfoot (M03), medial forefoot (M04), lateral forefoot (M05). The automatic geometry-based regionalization (GM) which best fitted the OFM definition was used for comparison: it is based on the bisecting line of the foot and on the 23% (hindfoot) and 55% (midfoot) perpendicular lines. Relevant baropodometric parameters were calculated for each footprint using AM and GM. To avoid smoothing effects due to intra-subject averaging, all available footprints were used and individually compared; non-parametric statistics was applied to all comparisons.

**Figure 1:**
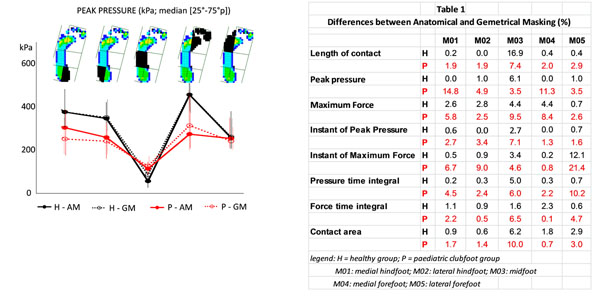
Median values and 25-75 percentile lines of peak pressure at each of the 5 plantar regions (black areas), obtained for the healthy population (H, black lines) and the paediatric clubfeet (P, red lines) from the anatomical masking (AM, solid lines) and the geometrical masking (GM, dotted lines).

## Results

143 healthy footprints and 84 clubfoot footprints (17 feet) were used in the study. Results from AM and GM were very similar for the healthy group, for all parameters and regions (median difference 0.9% [0.4-2.7]) except for midfoot length of contact and lateral forefoot instant of Maximum force; this proved that AM provides comparable results to GM in this population. Interestingly, the corresponding comparison applied to the pathologic group showed higher differences (3.4% [2.0-6.8]), despite the fact that most feet demonstrated near complete footprints.

## Conclusions

The proposed anatomical masking proved to be comparable to the corresponding geometrical masking on a large selection of healthy footprints. Differences between the two methods for clubfoot footprints suggested the appropriateness and the greater clinical relevance of the anatomical masking, which may better highlight changes in the loading pattern.
